# Trauma, experiences of sexual violence and mental health in LGBTIQ+ refugees seeking psychosocial support in Germany

**DOI:** 10.1017/S2045796025000216

**Published:** 2025-04-11

**Authors:** Yuriy Nesterko, Kim Schönenberg, Anna Weißig, Tatiana Kulbakina, Heide Glaesmer

**Affiliations:** 1 Department of Medical Psychology and Medical Sociology, University of Leipzig, Leipzig, Germany; 2 Department for Clinical Psychological Intervention, Free University Berlin, Berlin, Germany; 3 Department for Traumatic Stress and Transcultural Studies, Center ÜBERLEBEN, Berlin, Germany; 4 Queer Refugees Network, RosaLinde Leipzig e.V., Leipzig, Germany

**Keywords:** asylum-seekers, LGBTIQ+, refugee, sexual violence, trauma

## Abstract

**Aims:**

Only little empirical evidence exists on mental health in LGBTIQ+ refugees. In the present study, trauma exposure, experiences of sexual violence and current treatment needs for physical and mental health were investigated in association with symptoms of anxiety, depression, post-traumatic stress disorder (PTSD) and somatic symptom burden in LGBTIQ+ asylum-seekers resettled in Germany and seeking psychosocial support.

**Methods:**

Data was collected in cooperation with a counselling centre for LGBTIQ+ asylum-seekers between Mai 2018 and March 2024, with a total of 120 completed questionnaires of adult clients. The questionnaire (11 different languages) included sociodemographic and flight-related questions as well as standardized instruments for assessing PTSD (PCL-5), depression (PHQ-9), somatic symptom burden (SSS-8), and anxiety (HSCL-25). Prevalence rates were calculated according to the cut-off scores of each questionnaire. Four logistic regression analyses were conducted to test for potential associations between being screened positive for anxiety, depression, somatic symptom burden or PTSD and the number of traumatic events, experiences of sexual violence as well as current treatment needs for physical and mental health.

**Results:**

The great majority, 74.2% (95% CI: 66–82) of the respondents, screened positive for at least one of the mental disorders investigated, with 45% (95% CI: 36–54) suffering from somatic symptom burden, 44.2% (95% CI: 35–53) from depression, 58.3% (95% CI: 50–67) from PTSD, and 62.5% (95% CI: 54–71) from anxiety; 69.5% participants reported having been exposed to sexual violence. Current treatment needs for physical health problems were reported by 47% and for mental health problems by 56.7%. Participants with experiences of sexual violence were more likely to be screened positive for depression (OR: 6.787, 95% CI: 1.45–31.65) and PTSD (OR: 6.121, 95% CI: 1.34–27.95).

**Conclusions:**

The study provides initial insights on mental health and associated factors in a highly burdened and hard-to-reach population. The findings are important for healthcare systems and political authorities in terms of assuring better protection and healthcare for LGBTIQ+ refugees and asylum-seekers.

## Introduction

The number of people who are forced to leave their homes and become refugees is steadily increasing year on year, with estimates constantly reaching new and horrific records (United Nations High Commissioner for Refugees UNHCR, 2024). Within this group in urgent need of protection, there are particularly vulnerable populations, such as LGBTIQ+ refugees. In their countries of origin, many LGBTIQ+ refugees are forced to hide their sexual orientation and/or gender identity. As a result, many of them simply do not reveal their LGBTIQ+ identity to others in order to avoid legal and social restrictions. In a safe host country, LGBTIQ+ refugees are able to disclose; however, in many cases, they remain invisible and their mental healthcare needs are unmet (D’souza *et al.*, 2022). Similarly, the issue of invisibility applies to research on LGBTIQ+ refugees and asylum-seekers. For example, there is still limited evidence on the proportion of this subgroup within the European refugee population, even though a significant number of LGBTIQ+ refugees from the Middle East, North Africa and South Asia have applied for asylum in the European Union in recent years (Alessi *et al.*, 2018b). Furthermore, only little empirical evidence exists on mental health in this group (Bird *et al.*, 2024; White *et al.*, 2019).

Given the fact that they belong to many different minority groups in a society, LGBTIQ+ refugees are at a particularly high risk of experiencing adverse, potentially traumatizing events such as sexual violence (Hopkinson *et al.*, 2017; Piwowarczyk *et al.*, 2017), and, as a result, of developing severe mental health problems (Fox *et al.*, 2020). Those are frequently accompanied by insufficient access to and provision of mental healthcare (Kahn *et al.*, 2017; Lasowski *et al.*, 2023).

Based on theories of social stress, Meyer (2003) developed the concept of ‘minority stress’, as a result of recognizing that minorities (e.g. based on ethnicity, sexual orientation or gender identity) are exposed to additional or increased stress, differentiating between distal (external) and proximal (internal or internalized) stressors. Due to social structures and norms that often reject the realities of minority groups and thus can lead to prejudice and discrimination, minorities are exposed to increased social stress over a longer period of time or even to chronic stress during their lives. Discrimination and its various consequences represent distal stressors. Applied to LGBTIQ+ individuals, the following proximal stressors are discussed: (1) expected rejection by others, (2) society’s internalized negative attitude towards LGBTIQ+ individuals, (3) hiding one’s own sexual orientation and/or gender identity. The effects of these cumulative distal and proximal stressors on mental health are moderated by one’s own stress management capacities as well as social support. The concept can therefore provide an explanation for the higher prevalence rates of common mental disorders in minority populations compared to the general population (White *et al.*, 2019).

Looking at LGBTIQ+ refugees and asylum-seekers, an intersectional and cumulative impact should be expected: (1) due to non-conformity with the heteronormative expectations of host societies, (2) due to belonging to an ethnic minority, and (3) due to the legal status as a refugee or asylum-seeker. Other characteristics, such as skin colour, religious affiliation, and physical impairment, may act as additional stressors. It is important to emphasize that based on different affiliations and/or representations rejection and thus stress can be caused by representatives of different groups (e.g. majority society in the host country both in terms of racism and LGBTIQ+ hostility, LGBTIQ+ community in the host country excluding ethnic minorities, their own ethnic community following heteronormative norms and ideas, as well as other refugee and migrant groups expressing LGBTIQ+ hostile attitudes and behaviour and/or racism).

There is now clear evidence that refugees and asylum-seekers are particularly vulnerable to developing mental disorders (Giacco *et al.*, 2018; Nesterko *et al.*, 2020a). This is linked to a high risk of potentially traumatic experiences before, during and after fleeing or forced migration, but also to the political and social climate, legal framework in the respective host country after resettlement (Nesterko *et al.*, 2020b; Nickerson *et al.*, 2024). In addition, reliable evidence shows that LGBTIQ+ individuals are more likely to be affected by mental disorders than non-LGBTIQ+ individuals (Ploederl and Tremblay, 2015; Ross *et al.*, 2018), which can lead to increased rates of suicide, especially in adolescence and young adulthood, as suggested by recently published data on deaths by suicide among LGBTIQ+ youth in the US (Ream, 2022). As mentioned above, there are very few studies on trauma and mental health of LGBTIQ+ refugees, most of which use qualitative methods and report on refugees and asylum-seekers in North America (Alessi *et al.*, 2018a, 2018b; Bird *et al.*, 2024; Gowin *et al.*, 2017; Logie *et al.*, 2016). In a scoping review on mental health needs of LGBT refugees recently published by D’souza *et al.*’s (2022), 27 peer-review studies were included, with only four based on quantitative data. The key results from these and more recently published studies with a quantitative assessment of mental health in LGBTIQ+ refugees and asylum-seekers are summarized below.

In a study by Fox *et al.* (2020) with the largest sample to date (N = 308 LGBTIQ+ refugees, from 48 different countries, resettled to North America), 80.2% of the sample were screened positive for mental disorders with the Refugee Health Screener-15. The authors identified several factors predicting a positive screening result. As expected and consistent with the minority stress model, ‘loneliness’ (OR = 1.14) and ‘non-disclosure of LGBTIQ+ identity’ (OR = 3.46) each predicted significantly higher odds of being screened positive. Alessi *et al.* (2017) reported a prevalence of 64.9% for post-traumatic stress disorder (PTSD) (PCL-5 positive screen) in their study with 37 LGBTIQ+ refugees who had arrived in EU countries from the Middle East, North Africa and Asia. A similarly high prevalence rate was reported by Piwowarczyk *et al.* (2017) in their study among LGBTIQ+ refugees (N = 50) in the US: 70% of the sample were diagnosed with PTSD and 28% with anxiety disorder; in addition, 76% of respondents had a history of full/partial remission from major depression at the time of the interview or survey. Hopkinson *et al.* (2017) compared LGBTIQ+ refugees and non-LGBTIQ+ refugees (35 individuals each) regarding the frequency of experiencing sexual violence, revealing a significantly higher frequency of reports of sexual violence among LGBTIQ+ refugees, with perpetrators often being family members. In addition, LGBTIQ+ refugees reported significantly more suicidal thoughts. In a recently published study by Lasowski *et al.* (2023), high prevalence rates for sexual assault (56.1%), PTSD (83.3%), depression (72.7%), and anxiety (57.6%) were reported by a sample of 66 self-identified LGBTIQ+ asylum seekers from 24 different countries of origin being provided with healthcare services and forensic evaluations during their application for asylum in the US. In a study conducted in Berlin, Germany, among asylum seekers living in LGBTIQ+ shelters (n = 32) comparing them with residents of non-LGBTIQ+ shelters (n = 277), higher positive screenings for depression and/or anxiety (using PHQ-4) were reported for LGBTIQ+ shelter residents (70% vs. 34%), as well as higher use of outpatient mental health services (Gottlieb *et al.*, 2020).

Overall, the limited evidence primarily reflects the high symptom burden among LGBTIQ+ refugees and asylum-seekers, which may be attributed to an increased exposure to traumatic experiences, particularly experiences of sexual violence, resulting in an urgent need for mental healthcare. To the best of our knowledge, no data is available on traumatic experiences, and specifically on experiences of sexual violence, current treatment needs and symptoms of different mental disorders among LGBTIQ+ refugees in Germany. Therefore, the rationale of the present study was to investigate trauma exposure, experiences of sexual violence and current treatments needs for physical and mental health in association with symptoms of anxiety, depression, PTSD and somatic symptom burden in LGBTIQ+ refugees and asylum-seekers resettled in Germany and seeking psychosocial support. Based on data collected in a counselling centre for LGBTIQ+ asylum-seekers, the analyses presented below were guided by the following research questions:

1. What traumatic experiences do LGBTIQ+ asylum-seekers resettled in Germany report when seeking psychosocial support?
2. What are the prevalence rates for sexual violence, common mental disorders (PTSD, depression, anxiety, and somatoform symptom burden) and current treatment needs in LGBTIQ+ asylum-seekers resettled in Germany who are seeking psychosocial support?
3. How are trauma exposure, experiences of sexual violence and current treatment needs linked with symptoms of anxiety, depression, PTSD, and somatic symptom burdens?

## Methods

### Data collection and study sample

Data was collected jointly by the Department of Medical Psychology and Medical Sociology at Leipzig University and the Queer Refugees Network (QRN) of RosaLinde Leipzig e.V., a counselling centre for LGBTIQ+ asylum-seekers in Leipzig, Germany. For all clients, a basic data sheet was created listing socio-demographic and displacement-related characteristics assessed and filled out by the counsellors of QRN. From May 2018, screening questionnaires assessing common mental health symptoms were additionally used in order to assess traumatic experiences as well as symptoms of common mental disorders of the clients. The clients mostly completed the questionnaires independently. In case of need for assistance or questions, counsellors and translators were available to provide support.

Before the initial consultation, all clients were informed about data protection measures. It was verbally explained to the clients that the completed questionnaires would be individually evaluated by the counsellors and discussed with the clients as part of the ongoing consultation and would also be made available in a fully anonymized version to the Department of Medical Psychology and Medical Sociology of the University of Leipzig, if written informed consent was given by the client. All study procedures were conducted in accordance with the Helsinki Declaration and its later amendments or comparable ethical standards. Written informed consent was obtained from all study participants.

Between January 2016 and March 2024 socio-demographic characteristics of 250 clients have been collected by QRN counsellors. A total of 156 (62.4%) of these clients completed the questionnaire to assess common mental health symptoms as of May 2018. Of these 156 data sets 36 cases were incomplete because either information on sociodemographic characteristics or mental health outcomes was missing. Therefore, the present study analysed data collected between May 2018 and March 2024. A total of 120 questionnaires were completed by adult clients assessing symptoms of depression, anxiety, PTSD and somatic symptoms as primary outcomes.

### Instruments

The questionnaire used in the present study included sociodemographic and displacement-related questions, as well as standardized instruments for assessing symptoms of depression, PTSD and somatic symptoms. The German or English version of the questionnaire were translated and back-translated into 10 different languages (Albanian, Arabic, Farsi, French, Kurdish, Russian, Spanish, Tigrinya, Turkish and Urdu) by a professional translation agency specializing in medical translations. All back-translations were reviewed by the first and last author and, when necessary, returned to the agency for final modification/adjustment.

#### Sociodemographic and flight-related characteristics

Participants were asked to provide information about their age, sex (according to the entry in the official identification document or self-description), gender identity, sexual orientation, preferred pronouns, country of origin, parenthood, partnership, the duration of their flight/migration and the length of stay in Germany.

#### Self-rated treatment needs

Current treatment needs for mental and/or physical health conditions were assessed with two items (‘Do you currently need treatment for physical health problems?’/’Do you currently need treatment for mental/emotional health problems?’) with ‘yes’ or ‘no’ response options.

#### Traumatic events

Traumatic experiences were assessed using the revised DSM-5 Life Events Checklist (LEC-5) (Weathers *et al.*, 2013). The LEC-5 comprises 17 items, which address the experience of different types of events that can potentially result in PTSD or distress. The response option ‘happened to me’ (direct exposure) being the only one used in the present study.

#### Sexual violence

Experiences of sexual violence were assessed with two items from the DSM-5 LEC-5 (Weathers et al., 2013) ‘Sexual assault (rape, attempted rape, being forced to perform any type of sexual act by force or threat of force)’ and/or ‘Other unwanted sexual experiences’ with the response options ‘happened to me’ and/or ‘witnessed it’.

#### Posttraumatic stress disorder

PTSD was assessed with the PCL-5 (PTSD Checklist for DSM-5), a 20-item self-report instrument, which assesses symptoms of PTSD as defined by the DSM-5 (Blevins *et al.*, 2015), with each symptom rated on a 5-point Likert-scale ranging from ‘not at all’ (0) to ‘extremely’ (4). A total score (0–80) can be obtained by summing up the scores for each of the 20 items. A score at or above the cut-off score 33 is usually used to identify probable PTSD. Cronbach’s’ α in the present study was α = .94.

#### Depression

Symptoms of depression were assessed with the Depression module of the Patient Health Questionnare-9 (PHQ-9; Kroenke *et al.*, 2001). The PHQ-9 contains nine items rated on a scale of 0 (‘not at all’) to 3 (‘nearly every day’) which reflect the frequency with which participants have experienced the symptoms within the previous 14 days. Based on the total score (0–27), symptom severity can be divided into the categories ‘none-minimal’ (0–4), ‘mild’ (5–9), ‘moderate’ (10–14), ‘moderately severe’ (15–19), and ‘severe’ (20–27) depression. Participants with a total score of >14 were classified as having clinically relevant symptoms of depression. Cronbach’s α in the present study was α = .85.

#### Anxiety

Symptoms of anxiety were assessed with the anxiety-subscale of the Hopkins Symptom Checklist (HSCL-25; Derogatis *et al.*, 1974). The HSCL-25 anxiety-subscale consists of 10 items assessing symptoms experienced within the last week on a 4-point Likert-scale, (‘not at all’, ‘a little’, ‘quite a bit’, and ‘extremely’). As recommended by several studies that used the HSCL-25 in different refugee populations (Jakobsen *et al.*, 2011), individuals with a mean score of 1.75 or higher were classified as having clinically relevant symptoms of anxiety. The internal consistency of the anxiety-subscale in the study was α = .91.

#### Somatic symptoms

Somatic symptoms were assessed with the Somatic Symptom Scale-8 (SSS-8), a brief self-report measure of somatic symptom burden (Gierk *et al.*, 2014). Each of its 8 items can be rated on a 5-point Likert-Scale from ‘not at all’ (0) to ‘very much’ (4) referring to the previous 7 days. The total score therefore ranges from 0 to 32, and is subdivided into five categories of severity: ‘none to minimal’ (0–3), ‘low’ (4–7), ‘medium’ (8–11), ‘high’ (12–15), and ‘very high’ (16–32) somatic symptom burden. A cut-off-score of >11 was used for the present study to identify participants with clinically relevant somatic symptoms. The internal consistency was α = .85.

### Statistical analyses

Statistical analyses were performed using the IBM SPSS statistical package, version 27.0 for Windows. Descriptive statistics were used to characterize the study sample. Prevalence rates were calculated according to the cut-off scores of each questionnaire. Four logistic regression analyses were conducted to test for potential predictors for being screened positive for anxiety, depression, somatic symptom burden, and PTSD. For all four models the following potential predictor variables were analysed using enter method: age, experiences of sexual violence (dichotomized), number of traumatic events, and current treatment needs for physical and mental health.

## Results

Table 1 gives an overview of the sociodemographic and displacement-related characteristics of the sample. The mean age of the participants was 29.23 (SD = 7.44) years. The majority of the participants were male according to their official identification document or self-description (n = 72, 60%), 60.8% (n = 73) identified themselves as male in terms of gender identity, 47.5% (n = 57) preferred the pronouns he/him, and 78 (65%) participants stated being homosexual. The largest groups were participants from Venezuela (45.8%) and Cameroon (30.8%), followed by smaller groups of participants from Iraq, Nigeria and Pakistan: overall, participants from over 18 different countries took part in the survey. The mean length of stay in Germany was 7.1 months (SD = 10.2), reflecting a wide range from 0 to 60 months.

**Table 1.** Sociodemographic and displacement-related characteristics

	Participants, N = 120
**Age**	
M/SD/Range	29.23/7.44/18–56
**Sex (according to official identification document or self-description)**	
Male	72 (60%)
Female	19 (15.8%)
Other	4 (3.3%)
Not assessed	25 (20.8%)
**Gender identity**	
Male	73 (60.8%)
Female	24 (20%)
Other (including “both” and non-binary)	11 (9.2%)
Not assessed	12 (10%)
**Preferred pronouns**	
He/him	57 (47.5%)
She/her	24 (20%)
No pronouns	6 (5%)
Other	5 (4.2%)
Not assessed	28 (23.3%)
**Sexual orientation**	
Heterosexual	2 (1.7%)
Homosexual	78 (65%)
Bisexual	28 (23.3%)
Other (including pansexual and queer)	2 (1.7%)
Not assessed	10 (8.3%)
**Country of origin**	
Venezuela	55 (45.8%)
Cameroon	37 (30.8%)
Iraq	4 (3.3%)
Nigeria	3 (2.5%)
Pakistan	3 (2.5%)
Russian Federation	3 (2.5%)
Georgia	2 (1.7%)
Tunisia	2 (1.7%)
Other^a^	10 (8.3%)
Not assessed	1 (0.8%)
**Partnership**	
Yes	47 (39.2%)
No	46 (38.3%)
Not assessed	27 (22.5%)
**Parenthood**	
Yes	22 (18.7%)
No	77 (64.2%)
Not assessed	21 (17.5%)
**Lengths of stay in Germany in months**	
M/SD/Range	7.1/10.2/0–60

a Country of origin other (N): Afghanistan (1), Albania (1), Eritrea (1), Jordan (1), Kosovo (1), Lebanon (1), Libya (1), Myanmar (1), Serbia (1), Senegal (1).

**Figure 1.**
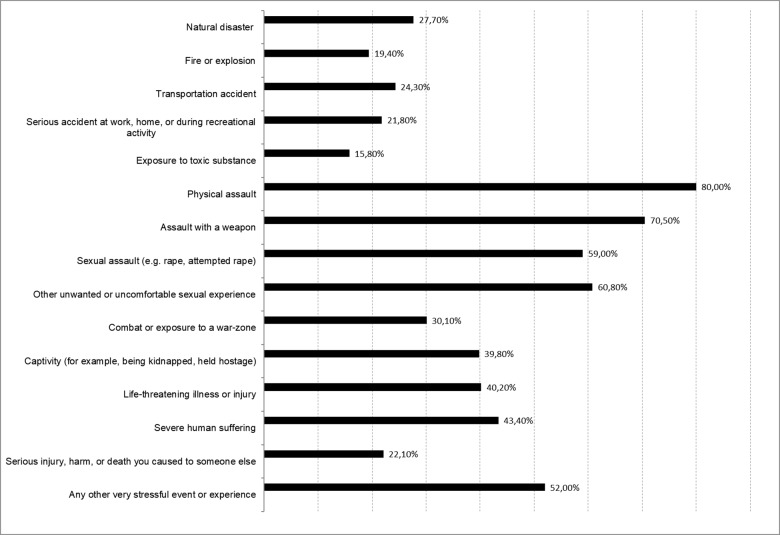
Traumatic events experienced by the study sample (N = 107).

The mean number of traumatic events reported by the participants was 5.84 (SD = 2.89, range 0–15) (Fig. 1). With 80% (n = 84/N = 105), physical assaults were the most frequently reported event, followed by assault with a weapon (70.5%; n = 74/N = 105), sexual assault (59%; n = 62/N = 105) and other unwanted or uncomfortable sexual experiences (60.8%; n = 62/N = 102). In total, 73 of 105 (69.5%) participants reported having been exposed to sexual violence. 105 of 107 participants (98.1%) reported having experienced at least one traumatic event.

Using established cut-off scores, prevalence rates of 45% (95% CI: 36–54) were identified for somatic symptom burden, 44.2% (95% CI: 35–53) for depression, 62.5% for anxiety (95% CI: 54–71), and 58.3% (95% CI: 50–67) for PTSD (Table 2). Current treatment needs for physical health problems were reported by 47% (95% CI: 38–56) and for mental health problems by 59.1% (95% CI: 50–68).

**Table 2.** Prevalence of anxiety, depression, somatic symptoms, and PTSD, number of traumatic events as well as self-rated treatment needs for physical and mental health problems

	N (%)	95% CI
**Somatic symptom burden**		
SSS-8 cut off >11	54 (45%)	36–54
**Depression**		
PHQ-9 cut-off >14	53 (44.2%)	35–53
**Anxiety**		
HSCL-25 (subscale Anxiety) cut-off >1.75	75 (62.5%)	54–71
**PTSD**		
PCL-5 cut-off >32	70 (58.3%)	50–67
**At least one mental disorder**		
Yes	89 (74.2%)	66–82
**Current treatment needs for physical health problems**^1^		
Yes	55 (47%)	38–56
**Current treatment needs for mental health problems**^2^		
Yes	68 (59.1%)	50–68
	**M/SD/Range**	
**Number of traumatic events**^3^	5.84/2.89/0–15	

1 N = 117.

2 N = 115.

3 N = 107.

**Figure 2.**
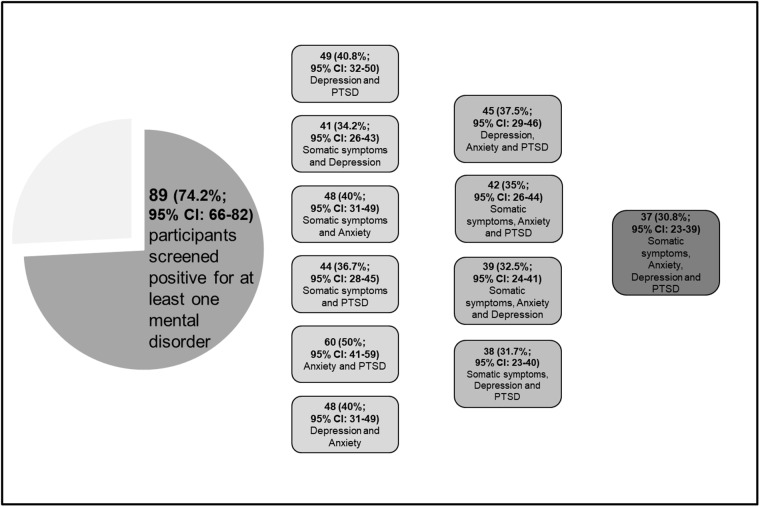
Comorbidity patterns of PTSD, anxiety, depression and somatic symptom burden in the study sample (N = 120).

74.2% (95% CI: 66–82) of the sample screened positive for at least one of the mental disorders assessed in the study according to the cut-off scores for the SSS-8, HSCL-25 (subscale Anxiety), PHQ-9 and PCL-5. The comorbidity patterns of mental disorders under study are shown in Figure 2.

Four separate logistic regression analyses were performed to test associations between experiences of sexual violence, number of traumatic events and current treatment needs for physical and mental health problems with anxiety, depression, PTSD and somatic symptom burden (Table 3). Participants with current treatment needs for mental health problems were more likely to be screened positive for anxiety (OR: 4.307, 95% CI: 1.63–11.40), depression (OR: 9.399, 95% CI: 2.98–29.64), and PTSD (OR: 10.694, 95% CI: 3.20–35.77); participants with current treatment needs for physical health problems were more likely to be screened positive for somatic symptom burden (OR: 6.153, 95% CI: 2.39–15.83) and depression (OR: 3.449, 95% CI: 1.22–9.76). Participants with experiences of sexual violence were more likely to be screened positive for depression (OR: 6.787, 95% CI: 1.45–31.65) and PTSD (OR: 6.121, 95% CI: 1.34–27.95). In addition, the number of traumatic events was found to be positively associated with PTSD (OR: 1.307, 95% CI: 1.03–1.66).

**Table 3.** Logistic regression analyses predicting anxiety, depression, somatic symptom burden and PTSD

	Anxiety			Depression			Somatic symptom burden			PTSD		
Predictor	Adjusted OR	95% CI	*p*	Adjusted OR	95% CI	*p*	Adjusted OR	95% CI	*p*	Adjusted OR	95% CI	*p*
Age	1.014	0.95–1.07	0.686	0.994	0.93–1.06	0.864	0.981	0.92–1.04	0.545	1.072	0.87–1.17	0.101
Experiences of sexual violence^a^	3.335	0.97–11.49	0.056	**6.787**	**1.45–31.65**	**0.015**	1.302	0.36–4.69	0.687	**6.121**	**1.34–27.95**	**0.019**
Number of traumatic events	1.012	0.84–1.22	0.895	0.998	0.82–1.21	0.981	1.069	0.90–1.27	0.456	**1.307**	**1.03–1.66**	**0.028**
Current treatment needs for physical health^a^	1.693	0.62–4.61	0.303	**3.449**	**1.22–9.76**	**0.020**	**6.153**	**2.39–15.83**	**<0.000**	1.323	0.43–4.10	0.628
Current treatment needs for mental health^a^	**4.307**	**1.63–11.40**	**0.003**	**9.399**	**2.98–29.64**	<**0.000**	2.223	0.82–6.03	0.117	**10.694**	**3.20–35.77**	**<0.000**
**Model fit indices:**												
χ^2^/df/*p*	22.970/5/<0.001			43.533/5/<0.001			26.523/5/<0.001			47.613/5/<0.001		
−2 Log-Likelihood	107.713			94.095			110.663			86.136		
Nagelkerkes R^2^	0.281			0.472			0.312			0.514		
Cox & Snell R^2^	0.205			0.353			0.233			0.379		

a Yes = 1; No = 0.

Bold values show significant predictors.

## Discussion

In the present study, trauma exposure, experiences of sexual violence, current treatment needs as well as prevalence of common mental disorders were investigated in LGBTIQ+ asylum-seekers resettled in Germany who are seeking psychosocial support. The results reveal high symptom burden in this population, with 74.2% screening positive for at least one of the four mental disorders investigated. The comorbidity rates are also high: almost a third (30.8%) screened positive for all four mental disorders, between 31.7% and 37.5% had clinically relevant symptoms of three different mental disorders, and 36.7% to 50% were found to have a clinically relevant symptom burden of two different mental disorders. The participants reported a mean of 5.84 different lifetime traumatic events, with physical assault (80%) and assault with a weapon (70.5%) being mentioned most frequently; experiences of sexual violence were reported by 69.5%. Finally, current treatment needs were reported by 47% for physical health problems and 56.7% for mental health problems. The findings are in line with previous research reporting high symptom burden and prevalence rates for common mental disorders in LGBTIQ+ refugees and asylum-seekers (Alessi *et al.*, 2017; Fox *et al.*, 2020; Gottlieb *et al.*, 2020) as well as a large number of traumatic events and experiences of sexual violence in this population (Hopkinson *et al.*, 2017; Lasowski *et al.*, 2023; Piwowarczyk *et al.*, 2017). Furthermore, when comparing these results with findings from a study among different groups of refugees in a primary reception facility in Leipzig, Germany, using the same methods, the prevalence of mental disorders among LGBTIQ+ asylum-seekers is higher than among refugees in general (e.g., prevalence rates for PTSD 58.3% vs. 34.7%, 62.5% vs. 41.8% for anxiety, 45% vs. 31.3% for somatic symptom burden, and 44.2% vs. 21.6% for depression; Nesterko *et al.*, 2020a, 2020b), supporting the evidence of a specifically high burden of mental disorders among LGBTIQ+ refugees and asylum-seekers. The results of the logistic regressions show associations between experiences of sexual violence and the likelihood for depression and PTSD – findings that are consistent with evidence on sexual violence and its mental health consequences (Khadr *et al.*, 2018; Oram, 2019; Sweeney *et al.*, 2019). In addition, the number of different kinds of traumatic experiences was found to be positively associated with PTSD, which is in line with previous research (Nesterko *et al.*, 2020a). Finally, current treatment needs for physical health problems were positively associated with clinically relevant symptom burden for depression and somatic symptoms, and current treatment needs for mental health problems were positively associated with anxiety, depression, and PTSD. Self-rated treatment needs may therefore be considered as an indicator for the presence of clinically relevant mental health problems in LGBTIQ+ refugees and asylum-seekers. Future research with larger samples is needed to investigate the topic in more detail. In general, the findings of this study should also be considered in light of the legal and social situation in the countries of origin of the participants. More than 75% of respondents originate from Cameroon (30.8%) and Venezuela (45.8%). In Cameroon, LGBTIQ+ individuals are persecuted by the state and violence can therefore be openly inflicted on them (Human Rights Watch, 2022). In Venezuela, LGBTIQ+ identities are not subject to prosecution, but there is no legal protection for LGBTIQ+ individuals, and they are socially stigmatized: for example, in 2013 71% of Venezuelans stated that homosexuality is morally unacceptable (Pew Research Center, 2014), and 32% opposed same-sex marriage in 2023 (Equilibrium CenDE, 2023).

The present study has some major strengths including (1) a relatively large sample size of a specific and hard-to-reach population, (2) assessment of trauma exposure, sexual violence and symptoms of four different mental disorders, (3) use of well-established instruments that were translated and back-translated into 11 different languages and (4) assessment of self-rated treatment needs, which had not been investigated in a comparable population before. However, there are a number of limitations requiring critical reflection. First, the sampling method is a limitation. As a convenience sample it might be biased, i.e. it only consists of clients of the QRN counselling centre. The following characteristics may have contributed to a bias: (1) all respondents are individuals who were able to navigate the care system to seek support and have therefore already managed to successfully overcome a major barrier in seeking help; in addition, those seeking help in a counselling centre might be suffering from particularly severe symptoms requiring them to seek support, (2) more than 60% of the sample identified as male and/or homosexual, and (3) as mentioned above, the great majority of the study sample had come from two countries, Venezuela and Cameroon. Second, the analyses conducted in the present study are based on cross-sectional data, thus no conclusions can be drawn regarding the direction of the associations observed as well as trajectories of the outcomes of interest. Third, due to the sample size and the heterogeneity of the sample with regard to their origin as well as sex, gender identity and/or sexual orientation, no comprehensive analyses on these socio-demographic characteristics were possible. Fourth, we had no comparison samples (e.g. refugees without an LGBTIQ+ background) or control groups (e.g. LGBTIQ+ individuals without a refugee background) that would have allowed a empirically derived conclusion about the cumulative and/or intersectional burden on the population under study. Consequently, the findings may not be generalizable to LGBTIQ+ refugees and asylum-seekers in Germany in terms of (1) assessments done in only one city, (2) specific proportion of countries of origin, (3) distribution of sex, gender identity and/or sexual orientation, (4) psychosocial support seeking of the sample, and (5) no direct comparisons with relevant controls. Future research is needed with a focus on mental health in LGBTIQ+ refugees and asylum-seekers assessing a broader spectrum of different LGBTIQ+ characteristics and using a longitudinal approach. Future studies should also take a closer look at the specific stressors that LGBTIQ+ refugees are confronted with, both when seeking psychosocial support and when not seeking it. Furthermore, studies are needed to identify the specific burdens faced by LGBTIQ+ refugees, also by comparing them with other relevant groups.

The main strength of this study is that it is the first study on traumatic experiences, sexual violence and mental health in LGBTIQ+ refugees and asylum-seekers in Germany based on quantitative data. Despite its exploratory nature, the study presents initial insights on mental health and associated factors in a highly burdened and hard-to-reach population. It provides impetus for further research and starting points for an in-depth examination of risk- and protective factors in LGBTIQ+ refugees and asylum-seekers. The findings clearly indicate the urgent need to inform healthcare providers, but also, more importantly, policy makers, about the specific needs of LGBTIQ+ refugees. This should aim at promoting the best possible integration of this population – in line with humanitarian protection obligations and the recognition of equal rights for all people regardless of their origin, sex, gender, sexual orientation, or mental and physical health.

## Data Availability

The data will not be shared due to ongoing analyses.
